# Diagnostic yield of allergy testing in Pediatric Eosinophilic Esophagitis: a 10 year experience at a tertiary care centre

**DOI:** 10.1186/1710-1492-10-S1-A28

**Published:** 2014-03-03

**Authors:** Perri R Tutelman, Jason Ohayon, Jefferson Terry, Mary E Sherlock

**Affiliations:** 1 Pediatric Gastroenterology and Nutrition, McMaster Children’s Hospital, McMaster University, Hamilton, Ontario, Canada; 2 Department of Pediatrics, McMaster Children’s Hospital, McMaster University, Hamilton, Ontario, Canada; 3 Department of Pathology and Molecular Medicine, McMaster Children's Hospital, McMaster University, Hamilton, Ontario, Canada

## Background

Eosinophilic Esophagitis (EoE) is a disease in children characterized by esophageal dysfunction and eosinophilic infiltration [1]. Patients frequently present with an atopic history; therefore, allergy testing by both skin prick and food patch testing are often performed. However, the utility of allergy testing remains unclear [1,3]. Treatment with topical corticosteroids may be needed for clinical and histologic improvement if allergic triggers are not identified [1,2]. However, the natural history of EoE suggests that this condition is chronic and may recur when corticosteroids are withdrawn [2]. Therefore, treatment with dietary/aeroallergen avoidance is preferable to reduce the need for oral corticosteroids, while preventing exacerbation of EoE [3]. This study aims to describe the atopic characteristics of a pediatric cohort with EoE over a 10 year period at a tertiary care center.

## Methods

All children (< 18 years) who had esophagogastroduodenoscopy with biopsy proven EoE (15+ eosinophils per high power field) at McMaster Children’s Hospital between January 2003 and December 2012 were identified and their medical records reviewed. Data pertaining to patient demographics, symptoms at diagnosis, atopic history, endoscopic and histologic findings, results of allergy testing (skin prick, patch and immunoCAP) and treatment outcomes were extracted.

## Results

**Figure 1:**
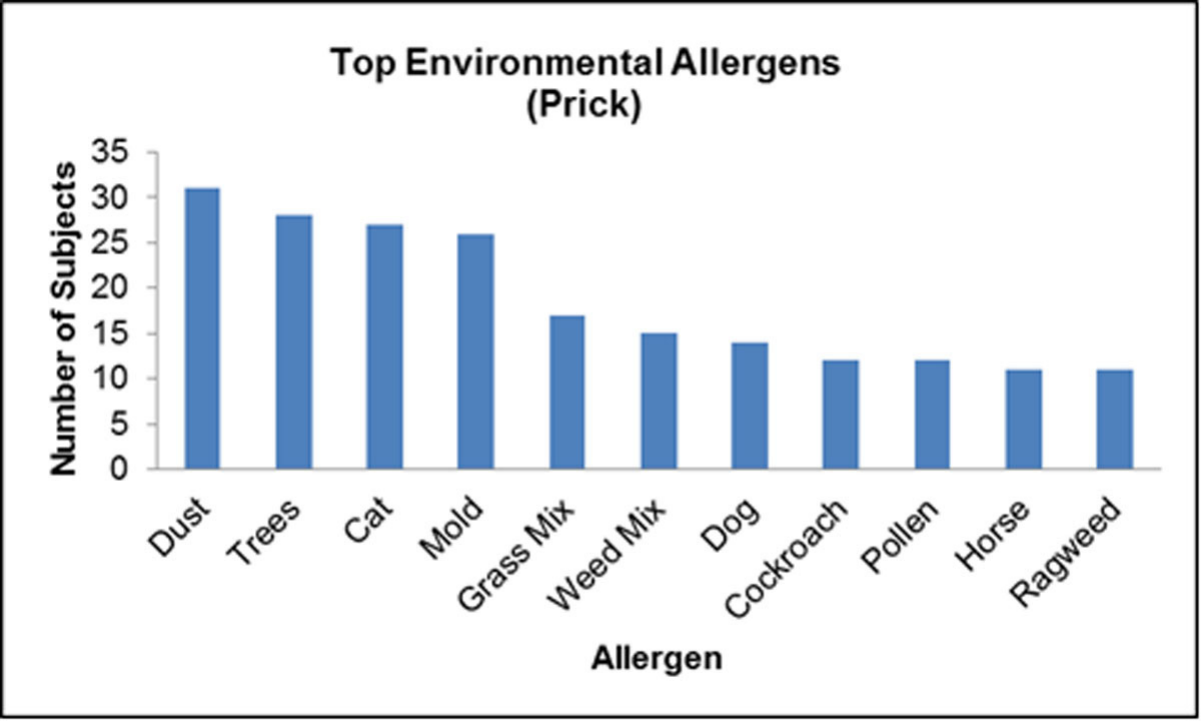
(N=69) underwent skin prick testing

**Figure 2:**
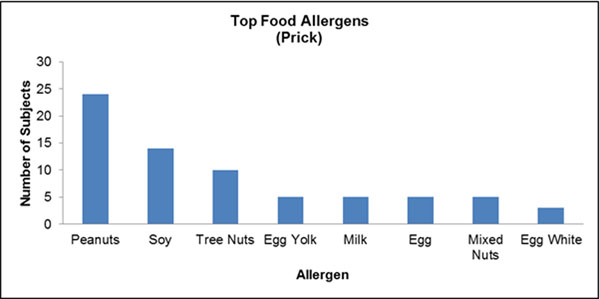
N=69 underwent skin prick testing

**Figure 3:**
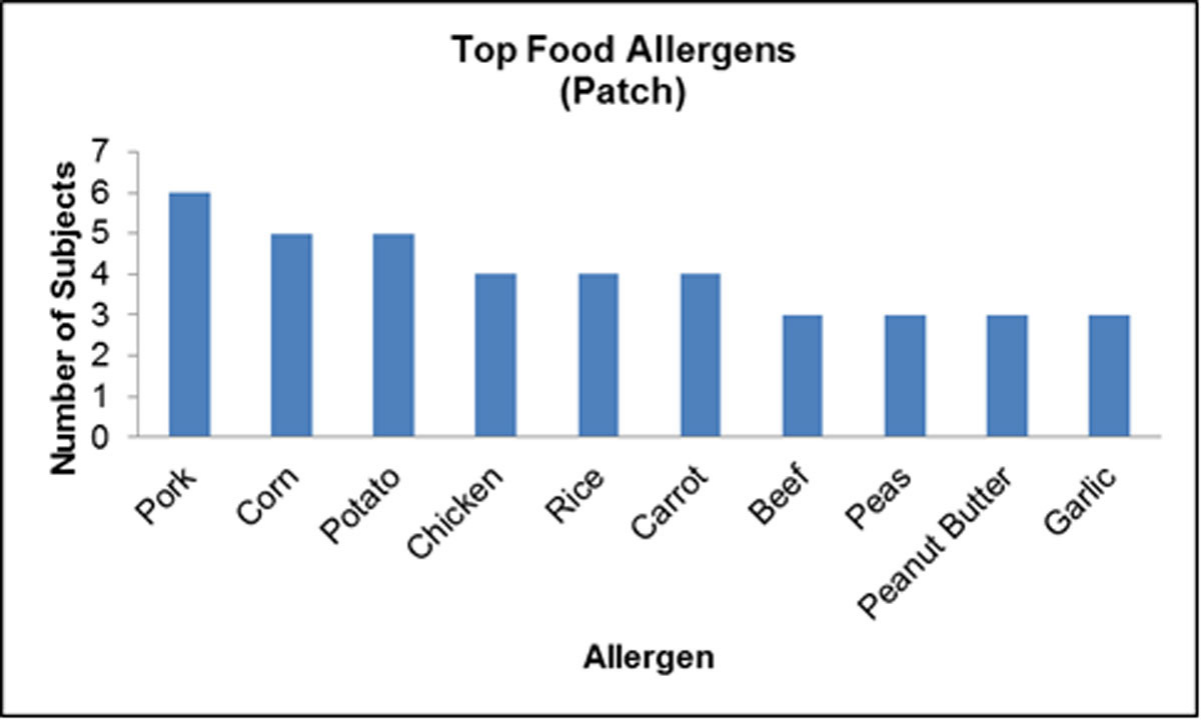
N=35 underwent patch testing

**Figure 4:**
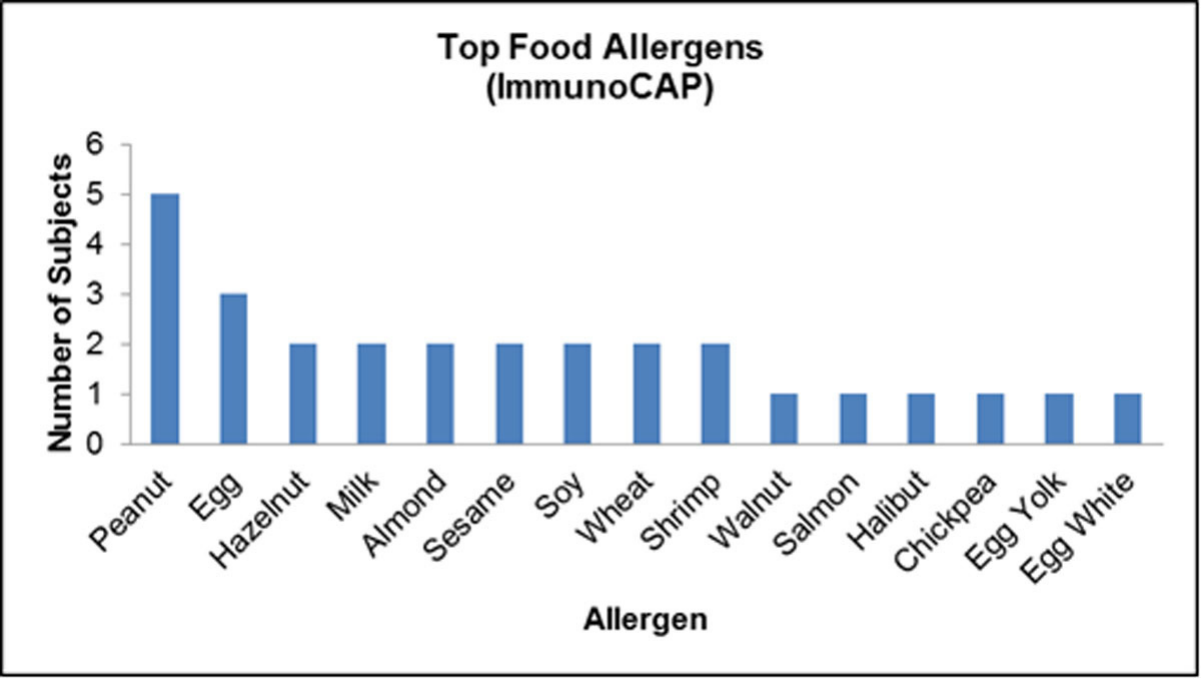
N=14 underwent ImmunoCAP testing.

A total of 96 cases were identified. The median age at diagnosis was 13.2 years (IQR=8.6-15.7), and 72 of the identified cases were male (74.2%). Seventy-four of 91 (81%) and 62 of 79 (78%) patients with data available reported a history of personal and family atopy, respectively. Six patients (7%) had a first degree family member diagnosed with EoE. Patients who reported a history of food allergy were significantly more likely to present with a food bolus impaction at diagnosis (p=0.035, OR=3.056). Sixty-nine patients underwent allergy testing as part of a standard EoE workup, of which, 60 (87.0%) had a positive test result. Ten (14.5%) patients who had identifiable allergies were positive only to environmental allergens. The top identified food and environmental allergens were recorded by allergy test type (Figs 1, 2, 3, 4).

## Conclusions

Pediatric EoE is considered to be an allergic disease with both environmental and food allergen triggers in many patients. A majority of patients present with a history of atopy and show positive allergy test results to both foods and aeroallergens. Both environmental and food allergen elimination should be considered a part of a therapeutic program. Identification of offending allergens by allergy skin and patch testing can be an important factor in guiding clinical decision making.
